# Identification of *SmNAC28* Transcription Factor and Its Mechanism of Regulating Salt Tolerance in Eggplant via S-Palmitoylation

**DOI:** 10.3390/cimb48040398

**Published:** 2026-04-14

**Authors:** Yuting Fan, Chenxiao Xu, Qi Chen, Wenhao Hu, Tuo Ji, Fengjuan Yang

**Affiliations:** 1 College of Horticulture Science and Engineering, Shandong Agricultural University, Tai’an 271018, China; fannyuting@163.com (Y.F.); xcx18853812186@163.com (C.X.); chihuoxiaochen@163.com (Q.C.); hu20000930@outlook.com (W.H.); 2 Key Laboratory of Biology and Genetic Improvement of Horticultural Crops in Huang-Huai Region, Ministry of Agriculture and Rural Affairs, Tai’an 271018, China; 3 Shandong Key Laboratory of Fruit and Vegetable Germplasm Innovation and Utilization, Tai’an 271018, China

**Keywords:** *Solanum melongena*, NAC transcription factor, salt stress tolerance, S-palmitoylation, post-translational modificatio

## Abstract

The NAC (NAM, ATAF1/2, and CUC1/2) family of transcription factors (TFs) play critical roles in regulating salt tolerance across diverse plant species. This study identified and characterized 101 NAC TFs in eggplant (*Solanum melongena* L.), revealing their diverse physicochemical properties, chromosomal distributions, and evolutionary relationships. Based on its salt stress-induced expression pattern and homology to known salt-responsive NAC factors, *SmNAC28* was selected as a key candidate for functional investigation of salt tolerance. Expression profiling indicated that *SmNAC28* is preferentially expressed in roots and stems, and its transcript levels are modulated by salt stress. Subcellular localization confirmed that *SmNAC28* localizes to both the plasma membrane and nucleus, a dynamic distribution regulated by S-palmitoylation. Under normal conditions, *SmNAC28* is anchored to the plasma membrane and nucleus via S-palmitoylation; upon salt stress exposure, it undergoes depalmitoylation and translocates to the nucleus. Using a hairy root transformation system in eggplant, we demonstrated that overexpression of *SmNAC28* in roots significantly enhanced salt tolerance by mitigating oxidative damage, maintaining ion homeostasis, and promoting osmotic adjustment. Analysis of transcript levels further revealed that *SmNAC28* overexpression upregulated ion transporter genes (*NHX2*, *CHXs*), signaling genes (*CIPKs*), and the proline biosynthesis gene (*P5CS*), which demonstrated that *SmNAC28* integrates antioxidant defense, ion homeostasis, and osmotic regulation to confer salt tolerance. This study reveals the response mechanism of *SmNAC28* to salt stress of the eggplant transcription factor *SmNAC28* under salt stress, and provided a research foundation for salt tolerance breeding.

## 1. Introduction

Eggplant (*Solanum melongena* L.) is an important vegetable crop in the Solanaceae family, widely cultivated in tropical and subtropical regions globally. In China, it ranks fourth in terms of protected cultivation area, valued for both its nutritional benefits and economic significance [1]. However, eggplant production frequently encounters abiotic stresses such as low temperature, drought, and soil salinity. These stresses often lead to growth inhibition, increased fruit malformation, and severe yield reduction, posing a major constraint to the sustainable development of the eggplant industry [2]. Consequently, identifying key genes that regulate stress resistance in eggplant and elucidating their molecular networks have become central research objectives for genetic improvement and breeding for stress tolerance.

NAC transcription factors (TFs), named after NAM, ATAF1/2, and CUC2, constitute plant-specific TF family [3]. They are characterized by a conserved NAC domain at the N-terminus, responsible for DNA binding and nuclear localization, and a more variable C-terminal transcriptional regulatory domain involved in activating or repressing target genes [4]. Some NAC proteins also possess a transmembrane motif, allowing them to remain membrane-associated and inactive until activated by specific stimuli, representing an important layer of post-translational regulation [3]. The conserved N-terminal domain combined with a divergent C-terminal region enables the functional diversification of NAC transcription factors across various plant biological processes, including development, senescence, and stress responses [5].

In response to salt stress, NAC TFs play multifaceted regulatory roles through mechanisms encompassing osmotic adjustment, reactive oxygen species (ROS) scavenging, phytohormone signaling integration, and the regulation of stress-responsive genes [6]. Salt stress causes a decline in cellular osmotic potential. NACs can enhance osmotic tolerance by promoting the synthesis of osmolytes such as proline and soluble sugars, thereby helping maintain water balance. For instance, rice *ONAC022* improves salt tolerance by increasing proline and soluble sugar content while reducing water loss and transpiration rate [7]. Similarly, *Suaeda liaotungensis SiNAC10* enhances salt resistance by binding to the promoters of proline biosynthesis genes and positively regulating their transcription [8]. Regarding ROS homeostasis, NACs mitigate oxidative damage by modulating ROS metabolism. Soybean *GmSIN1* amplifies the salt stress signal by binding to the promoters of *GmNCED3s* and *GmRbohBs*, promoting ABA and ROS accumulation [9]. Rice *OsNAC2* participates in salt stress-induced programmed cell death by regulating the expression of the ROS-scavenging gene *OsCOX17* and the caspase-like protease gene *OsAP37* [10]. Furthermore, NACs influence salt tolerance via hormone signaling pathways. Sweet potato *IbNAC3* enhances salt tolerance by transactivating *ERA1*, a negative regulator of ABA signaling, thereby reducing ABA sensitivity [11]. In *Arabidopsis*, *NTL8* is upregulated under high salinity and delays seed germination by suppressing gibberellin biosynthesis [12]. NACs also directly regulate the expression of stress-responsive genes. Soybean *GmNAC109* improves salt tolerance by modulating genes like *DREB1A*, *DREB2A*, and *AREB1* [13], while chrysanthemum *DNAC1* enhances salt resistance by regulating stress-responsive genes such as *KIN1* and *AMY1* [14]. Notably, some NACs are involved in salt-avoidance tropism; for example, the *Arabidopsis* root cap NAC TF SOMBRERO (SMB) guides roots away from high-salt environments by influencing auxin distribution in the lateral root cap and the expression of the auxin influx carrier gene *AUX1* [15]. Collectively, NAC TFs enhance plant adaptation to salt stress through a coordinated multilayered network involving osmotic adjustment, ROS homeostasis, hormonal crosstalk, and stress gene regulation, highlighting their significant potential for improving crop salt tolerance via genetic engineering.

Although extensive studies have established the critical roles of NAC TFs in plant salt stress responses at the transcriptional level [16,17], the dynamic subcellular regulation and post-translational control of NAC proteins remain largely underinvestigated. Increasing evidence indicates that transcription factor activity is not only governed by transcript abundance but also tightly modulated by post-translational modifications (PTMs) [18,19], which enable rapid and flexible responses to environmental signals. S-palmitoylation is a key reversible protein lipid modification, involving the covalent attachment of long-chain fatty acids to cysteine residues. This modification influences protein membrane association, subcellular localization, stability, and proteinprotein interactions [20,21]. Recent studies have shown that S-palmitoylation is widely involved in plant stress signaling. For instance, the NAC TF *MfNACsa* in *Medicago falcata* is anchored to the plasma membrane via S-palmitoylation. Under drought stress, it is depalmitoylated by the thioesterase APT1, translocates to the nucleus, and activates downstream stress-responsive genes, demonstrating the crucial role of the palmitoylation cycle in NAC-mediated stress responses [22]. Another study identified that *OsDHHC13* palmitoylates the rice NAC TF *OsNAC9*, and this modification regulates its plasma membrane localization, suggesting conservation of palmitoylation among NAC family members [23]. However, in eggplant and other Solanaceae crops, whether and how S-palmitoylation regulates NAC protein subcellular localization and salt tolerance remains unknown, representing a significant knowledge gap.This study accomplished genome-wide identification and bioinformatic analysis of the NAC family in eggplant, and screened the key gene *SmNAC28* through expression profiling and co-expression networks under salt stress. Functional investigation demonstrated that overexpression of *SmNAC28* systemically activates the expression of downstream salt-tolerance-related target genes, thereby significantly enhancing plant salt tolerance. Further mechanistic analysis revealed that this gene dynamically regulates its membrane-nucleus shuttling via S-palmitoylation modification in response to salt stress signals. This research provides a theoretical foundation and genetic resources for elucidating the molecular mechanisms of salt tolerance in Eggplant.

## 2. Materials and Methods

### 2.1. Identification and Chromosomal Distribution of NAC Genes in Eggplant

The eggplant genome sequence(https://solgenomics.net/ftp/genomes/Solanum_melongena_V4.1/Eggplant_V4.1.fa, accessed on 8 December 2025), protein database (https://solgenomics.net/ftp/genomes/Solanum_melongena_V4.1/Eggplant_V4.1_protein.function.fa, accessed on 8 December 2025), and annotation files corresponding (https://solgenomics.net/ftp/genomes/Solanum_melongena_V4.1/Eggplant_V4.1_repeats.gff3, accessed on 8 December 2025) to the Eggplant genome consortium V4.1 version were retrieved from the Solanaceae Genomics Network (https://solgenomics.net/, accessed on 8 December 2025). The Hidden Markov Model (HMM) file for NAM domain (PF02365) was downloaded from Pfam database (https://pfam.xfam.org/, accessed on 8 December 2025), and was used to retrieve the NAC proteins with a cut-off value of 0.001 by HMMER 3.3.2 (HHMI Janelia Research Campus, Ashland, VA, USA) (http://hmmer.org/download.html, accessed on 8 December 2025). BLASTP (basic local alignment search tool for proteins) against *S. melongena* genome data with *A. thaliana* NAC protein sequences retrieved from The *Arabidopsis* Information Resource (TAIR, https://www.arabidopsis.org/, accessed on 10 December 2025) was implemented (*e*-value = 0.001). Taking these two results together, the final members of the *SmNAC* genes were acquired and verified by PfamScan (*e*-value = 0.001, https://www.ebi.ac.uk/Tools/pfa/pfamscan/, accessed on 12 December 2025) and NCBI’s conserved domain database (NCBI-CDD, *e*-value = 0.001, https://www.ncbi.nlm.nih.gov/cdd/, accessed on 12 December 2025). The redundant sequences and sequences without a NAM domain were removed from the dataset. The basic information for *SmNAC* gene, including chromosome localization, intron number, average intron length, protein length, and isoelectric point (*pI*) values was determined based on the genome database. The chromosomal distribution map of *SmNAC* genes was presented and visualized using TBtools (South China Agricultural University, Guangzhou, Guangdong, China) (https://github.com/CJ-Chen/Tbtools, accessed on 18 December 2025).

### 2.2. Phylogenetic Analysis of NAC Proteins

The amino acid sequences of the NAC members of *S. melongena* and *A. thaliana* were aligned using Clustal X (University College Dublin, Dublin, Ireland), and a neighbor-joining unrooted phylogenetic tree with 1000 bootstrap replications was constructed by MEGA 7.0 (Molecular Evolutionary Genetics Analysis, Tempe, AZ, USA) (www.megasoftware.net, accessed on 15 December 2025). Finally, the tree was further modified by iTOL v6.5.8 (https://itol.embl.de/, accessed on 15 December 2025).

### 2.3. Gene Structure, Motif Identification, and Collinearity Analysis

The intron/exon structure of *SmNAC* genes was determined with the online gene structure display server (http://gsds.gao-lab.org/, accessed on 18 December 2025). The conserved motifs in *SmNAC* proteins were identified by MEME suite v5.4.1 (University of Nevada, Reno, NV, USA) (http://meme-suite.org/, accessed on 18 December 2025). The collinearity relationship of the *S. melongena* NAC genes between *A. thaliana* [24] and *S. lycopersicum* [25] were analyzed by MCScanX (University of Georgia, Athens, GA, USA) (https://github.com/wyp1125/MCScanX, accessed on 18 December 2025). These results were presented and visualized using TBtools (https://github.com/CJ-Chen/Tbtools, accessed on 18 December 2025).

### 2.4. Protein Tertiary Structure Prediction

The tertiary structures of eggplant NAC proteins were predicted using the AlphaFold Protein Structure Database (DeepMind, Islington, London, UK) (https://alphafold.com/, accessed on 25 December 2025). For the *SmNAC28* model, prediction confidence at each residue was evaluated by the predicted Local Distance Difference Test (pLDDT): pLDDT > 90 indicates very high confidence, 70–90 indicates confident regions, 50–70 indicates low confidence, and <50 indicates very low confidence. The overall model quality was assessed using the pTM score. The predicted positional errors between residues were analyzed using the Predicted Aligned Error (PAE) heatmap.

### 2.5. Expression Analysis of NAC Genes in Response to Salt Stress

Public RNA-seq datasets PRJNA649852 and PRJNA296071 (https://www.ncbi.nlm.nih.gov/bioproject, accessed on 20 December 2025) were retrieved from the NCBI Sequence Read Archive (SRA) to profile the expression of NAC family genes in *Arabidopsis thaliana* and *Oryza sativa* under salt treatment. Reads per kilobase per million mapped reads (FPKM) values were extracted and used to assess transcript abundance. Expression heatmaps were generated with TBtools (v2.0) to visualize differential expression patterns across samples and treatments.

### 2.6. Plant Materials and Treatment

Eggplant seedlings (*Solanum melongena* L. cv. ‘Baishui’) were cultivated under controlled conditions (28°C/18°C). For salt stress treatment, one-month-old seedlings with uniform growth were selected and treated with 100 mM NaCl in 1/2 Hoagland solution [26]. Samples were collected at 0, 1, 3, 6, and 12 h, as well as at 1, 2, 3, 4, 5, 6, and 7 days post-treatment. For tissue-specific expression analysis, six-month-old mature plants (reproductive stage) were used, and various tissues including roots, stems, leaves, flowers, fruits, and seeds were collected. All samples were flash frozen in liquid nitrogen and stored at −80°C until RNA extraction. Three biological replicates were performed for each treatment and tissue type, with each replicate representing an individual plant.

### 2.7. Quantitative Real-Time PCR Assays

Total RNA was extracted from the root of eggplant seedlings using an RNA extraction kit (Vazyme, Beijing, China). Quantitative real-time PCR (qRT-PCR) assays were conducted, as described previously [27], using an ABI PRISM 7500 Real-time PCR System (Applied Biosystem, Foster City, CA, USA) with 2^−ΔΔCT^ method [28]. The specific primers of *SmNAC* genes used here are listed in Table S1. The eggplant gene actin (*Smactin*) was used as an internal control.

### 2.8. Subcellular Localization Analysis

Potential palmitoylation sites in *SmNAC28* were predicted using the CSS-Palm 4.0 platform (Central South University, Changsha, Hunan, China). The *SmNAC28* sequence was cloned and subjected to site-directed mutagenesis, and then inserted into the pCAMBIAsuper1300-GFP vector respectively. The (PM) marker *AtCBL9* (AT5G47100.1) [29] and the nucleus (NC) marker *AtH3* (AT1G01370) [30] were used as references. The obtained plasmids were transformed and then inoculated into *Agrobacterium tumefaciens* GV3101, and the transformed *Agrobacterium tumefaciens* was subsequently injected into tobacco plants for transient expression. Under normal conditions, the transformants were directly observed using a confocal laser scanning microscope after cultivation. In the salt stress treatment, the transformants were cultured in 100 mM NaCl for 4 h before observation. For the hydroxylamine (NH_2_OH) treatment, the transformants were soaked in 0.05 M NH_2_OH for 2 h before observation. Subcellular localization was detected and imaged using a laser scanning confocal microscope (Zeiss LSM880, Jena, Germany).

### 2.9. Transcriptional Activation Assay

The full-length and truncated CDS of *SmNAC28* were cloned into the pGBKT7 vector, respectively. Each construct was co-transformed with the empty AD vector into the yeast Y2H strain. Transformants were plated on SD/-Leu/-Trp and SD/-Leu/-Trp/-His/-Ade+X-α-Gal media. Interactions were observed after 3 days of incubation at 30 °C.

### 2.10. Generation of Composite Eggplant Plants via Hairy Root Transformation

Four-week-old eggplant seedlings with uniform growth were selected. The primary roots were aseptically excised, and the wounded stem bases were immersed in a suspension of *Agrobacterium rhizogenes* strain K599 for 15 min [31]. Seedlings inoculated with K599 carrying the empty vector served as the negative control. After infection, plants were embedded in moist vermiculite, covered to maintain high humidity, and co-cultivated in darkness at 28 °C for 3 days. They were then transferred to a growth chamber (28 °C, 16 h light/8 h dark) and irrigated daily with 1/2 Hoagland nutrient solution to induce hairy root growth.

### 2.11. Determination of Phenotypes and Physiological Indices Under Salt Stress

Uniform *SmNAC28*-overexpressing (OE) and empty vector control (VC) eggplant seedlings at the four-leaf stage were hydroponically cultured and acclimated for 2 d. Seedlings were then subjected to either 100 mM NaCl treatment or maintained under wild-type (WT) control. For phenotypic observation, 14 biological replicates (*n* = 14) were used, while for physiological index measurements, three biological replicates (*n* = 3) were employed, with each biological replicate consisting of at least three individual seedlings pooled for sampling. After 6 d of treatment, samples were collected for phenotypic observation, including leaf wilting, leaf color, and root length.

A variety of physiological indices were determined in this study. Malondialdehyde (MDA) content was measured by the thiobarbituric acid (TBA) colorimetric method [32], with values expressed as μmol·g^−1^ FW. Antioxidant enzyme activities were analyzed using corresponding methods, with superoxide dismutase (SOD) activity was detected by the nitroblue tetrazolium (NBT) photoreduction method (U·g^−1^ FW), peroxidase (POD) activity by the guaiacol method (ΔA470·min^−1^·g^−1^ FW), and catalase (CAT) activity by the ultraviolet absorption method (ΔA240·min^−1^·g^−1^ FW) [33]. After sample digestion, Na^+^ and K^+^ contents were measured using a flame photometer and presented as mg·g^−1^ DW, and proline content was quantified by acidic ninhydrin colorimetry [34] and recorded as μg·g^−1^ FW.

### 2.12. Statistical Analysis

All statistical analyses were performed using SPSS 17.0 software (SPSS Inc., Chicago, IL, USA), and all figures were plotted using GraphPad Prism 10.1.2 software (GraphPad Software, San Diego, CA, USA). For comparisons between two groups, a two-tailed Student’s *t*-test was used; for comparisons involving three or more groups, one-way analysis of variance (ANOVA). All data are presented as mean ± SD from three biologically independent replicates, each originating from an individual plant or an independent transformation event (*n* = 3). Statistical significance was defined as * *p* < 0.05, ** *p* < 0.01, *** *p* < 0.001, and **** *p* < 0.0001.

## 3. Results

### 3.1. Identification of NAC TFs in Eggplant

A total of 101 genes were identified, all of which contained a NAM domain (PF02365) specific to the NAC transcription factor family. Based on their chromosomal locations, they were designated as *SmNAC1* to *SmNAC101*. We analyzed the physicochemical characteristics of SmNAC proteins, including amino acid number, molecular weight, isoelectric point (pI), instability index, and subcellular localization. The length of the eggplant NAC proteins ranged from 63 aa (*SmNAC74*) to 1148 aa (*SmNAC85*), with molecular weights varying from 7618.73 Da (*SmNAC74*) to 126691.6 Da (*SmNAC85*). The instability index values spanned from 20.54 (*SmNAC82*) to 79.07 (*SmNAC74*), and the grand average of hydropathicity (GRAVY) ranged from −1.188 (*SmNAC75*) to −0.296 (*SmNAC92*). The isoelectric point analysis revealed that 66 members had a pI less than 7, while 35 members had a pI greater than 7, suggesting their potential functional roles under different physiological condition. Subcellular localization predictions indicated that 82 *SmNAC* members were localized in the nucleus, with a few distributed in other organelles such as the cytoplasm, chloroplasts, and peroxisomes (Table S2).

### 3.2. Phylogenetic Analysis of NAC Transcription Factors

**Figure 1:**
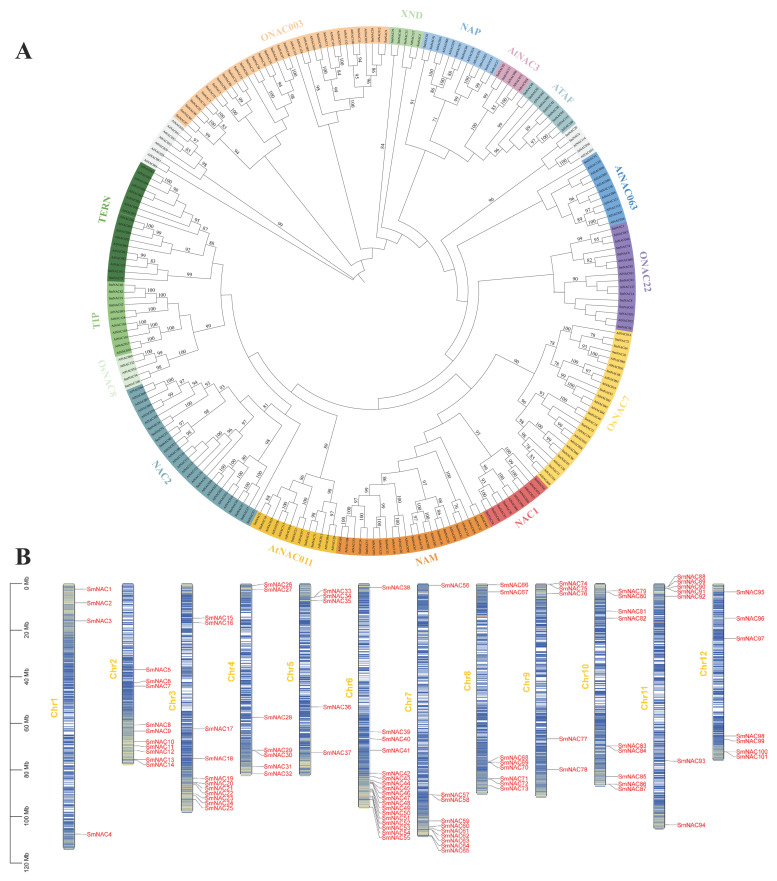
Phylogenetic and chromosomal distribution analyses of *SmNAC* gene family. (**A**) Phylogenetic relationships and subgroup designations of NAC transcription factors of *S. melongena* and *A. thaliana*; (**B**) physical map of 101 *SmNAC* genes on 12 chromosomes.

To investigate the phylogenetic relationships of *SmNAC* transcription factors, a phylogenetic tree was constructed using the Neighbor-Joining (NJ) method. Based on the NAC subfamily classification established for *Arabidopsis thaliana*, these proteins were categorized into 15 distinct subfamilies [35]. Within the eggplant NAC protein sequences, the ONAC003 subfamily contained the highest number of NAC members (25), followed by the OsNAC7 subfamily with 11 members. The OsNAC8 and AtNAC3 subfamilies had the fewest members, each containing only two members (Figure 1A).

Chromosomal distribution analysis revealed significant clustering patterns (Figure 1B). The *SmNAC* genes were unevenly distributed across the chromosomes, predominantly localized at the subtelomeric regions. Among these, chromosome chr6 harbored the highest number of *SmNAC* genes, with a total of 18 copies; these were predominantly located in subtelomeric regions, consistent with rapid evolution in repeat-rich, recombination-prone regions.

### 3.3. Gene Structure and Conservation Motif Analysis

**Figure 2:**
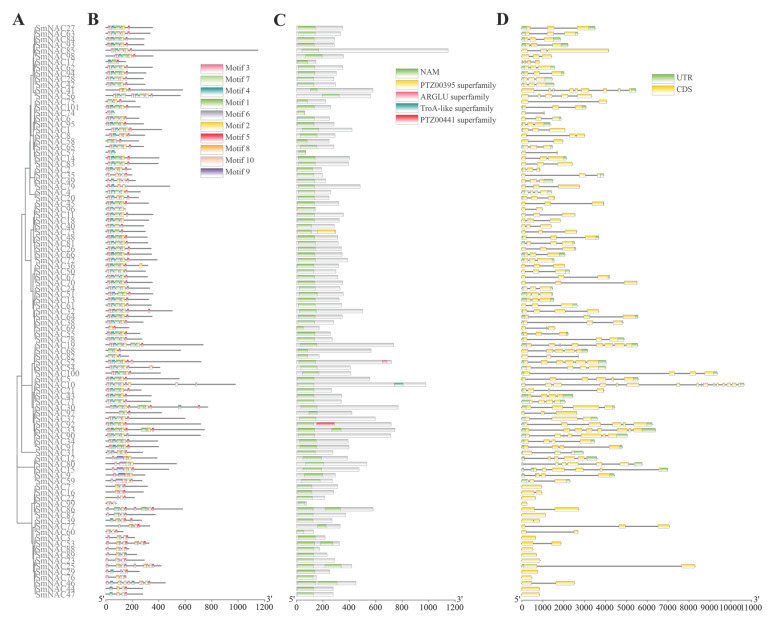
Structural analysis of *SmNAC* genes. (**A**) Phylogenetic tree of *SmNAC* proteins. (**B**) Distribution of conserved motifs identified by MEME. (**C**) Conserved NAC domain architecture predicted by SMART. (**D**) Exon–intron structures of the *SmNAC* genes.

To elucidate the structural diversity of the *SmNAC* members, conserved motifs, domains, and gene structures were visualized in conjunction with the phylogenetic tree, facilitating a comparative analysis of their evolutionary relationships and structural features (Figure 2A). Conserved motif analysis of the *SmNAC* members was performed using MEME, which identified ten distinct motifs (Figure S1). Most NAC members contained five motifs (Motif 3, Motif 7, Motif 4, Motif 1, and Motif 6), which constitute the highly conserved N-terminal portion of NAC proteins (Figure 2B), suggesting potential simplification or divergence of functional modules. Using TBtools, we identified conserved domains within the *SmNAC* genes. All *SmNAC* genes were found to contain the NAM-specific domain, and members of the PTZ00395, PTZ00441, ARGLU, and TroA-like superfamilies were also identified (Figure 2C). Gene structure analysis (Figure 2D) revealed that among the 101 *SmNAC* genes, 10 members were intronless, while the remaining members exhibited an exon–intron structure. Genes within the same subgroup often displayed similar gene structures, indicating a conserved exon–intron organization among the *SmNAC* genes.

### 3.4. Syntenic and Evolutionary Patterns of NAC TFs

**Figure 3:**
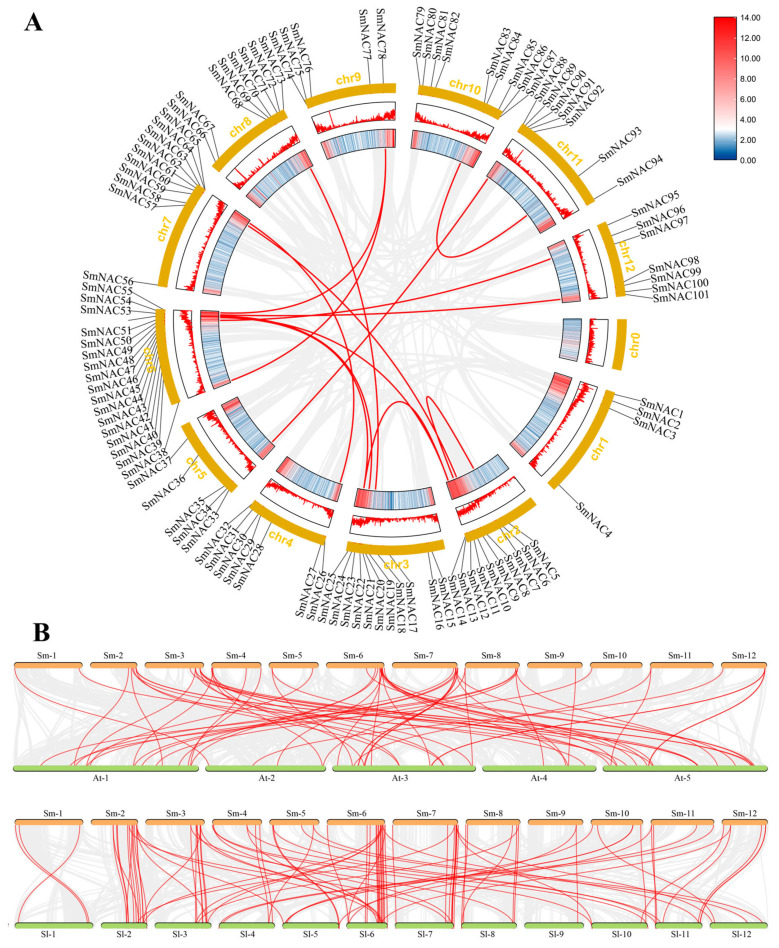
Collinearity analysis of NAC genes. (**A**) Intra-genomic collinearity within the *S. melongena* genome. Red curves connect segmentally duplicated gene pairs. (**B**) Interspecific synteny analysis between *S. melongena* and two related species (*S. lycopersicum* and *A. thaliana*). Grey lines in the background represent collinear blocks, while red lines highlight syntenic NAC gene pairs.

Gene duplication events are closely associated with the expansion of gene families. In this study, BLASTP and MCScanX tools were employed to analyze duplication events within the *SmNAC* gene family. We identified 13 segmentally duplicated gene pairs and one tandemly duplicated gene pair (*SmNAC5/10*) among the *SmNAC* family members (Figure 3A, Table S3). These 13 segmental duplication pairs and one tandem duplication pair were distributed across all 11 chromosomes except chromosome 1 (Figure 3A). Furthermore, we calculated synonymous (Ks) and non-synonymous (Ka) substitution rates to investigate the selective pressures acting on these homologous gene pairs, aiming to understand the expansion of this gene family in eggplant (Table S3). The Ka/Ks ratio for the tandemly duplicated *SmNAC* gene pair was 0.265. The Ka/Ks ratios for the segmentally duplicated gene pairs ranged from 0.113 to 0.419, with an average of 0.233. All Ka/Ks ratios for both tandemly and segmentally duplicated *SmNAC* gene pairs were less than 1, indicating that these genes have evolved under the influence of purifying selection. The average Ka/Ks value of tandemly duplicated genes (0.265) was higher than that of segmentally duplicated genes (0.233), suggesting that tandem duplicates evolved faster than other duplication events. The duplication time of *SmNAC* homologous pairs was estimated using the relative Ks metric as a proxy for time, with a time span ranging from 16.939 to 114.453 million years ago (MYA) and an average duplication time of 43.053 MYA. To further investigate the evolutionary relationships of the NAC gene family in eggplant, interspecific synteny analysis was performed. This analysis identified 65 and 92 homologous gene pairs between eggplant and *Arabidopsis thaliana*, and between eggplant and tomato, respectively (Figure 3B). These results indicate a close association between syntenic relationships and evolutionary divergence.

### 3.5. Transcriptional Expression Pattern Analysis of NAC Genes in Response to Salt Stress

**Figure 4:**
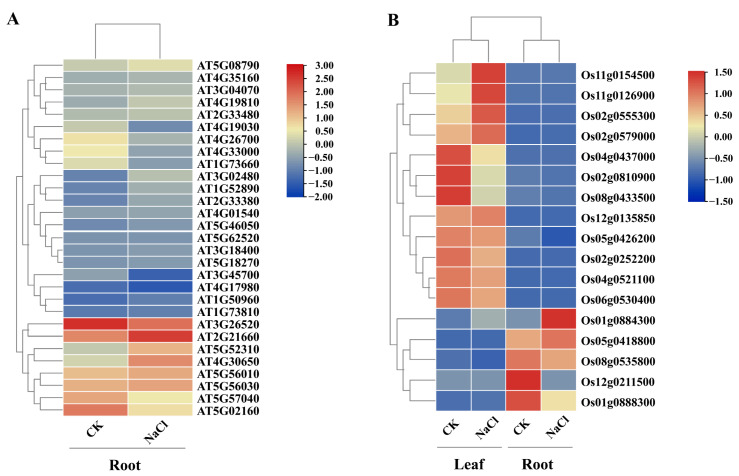
Expression heatmap of NAC family genes under salt stress. (**A**) *Arabidopsis thaliana* NAC family genes. (**B**) *Oryza sativa* NAC family genes.

Based on public transcriptome data from the NCBI SRA database, we further analyzed and visualized the expression levels of *NAC* family genes in *Arabidopsis thaliana* and *Oryza sativa* under salt stress using FPKM values (Figure 4). In *Arabidopsis*, a total of 29 *NAC* transcription factors were differentially expressed in roots after salt treatment, among which key genes such as AT5G08790, AT2G21660, AT4G30650, and AT5G52310 showed pronounced up-regulation. In rice, 16 genes were involved in the salt stress response following treatment. Genes including Os11g0154500, Os02g0555300, Os02g0579000, Os12g0135850, and Os11g0126900 were down-regulated in leaves but up-regulated in roots under salt stress, whereas Os01g0884300, Os02g0579000, and Os02g0555300 were up-regulated in both leaves and roots. Notably, AT5G08790 in *Arabidopsis* and Os01g0884300 in rice are orthologous genes; both exhibited consistent up-regulation under salt stress and belong to the core responsive members in their respective species. This evolutionarily conserved up-regulation pattern, together with their high sequence homology with *SmNAC28* in eggplant, strongly suggests that *SmNAC28* likely plays a conserved role in salt stress response in eggplant. Therefore, to further elucidate the functional mechanisms of NAC transcription factors in abiotic stress adaptation in eggplant, we selected *SmNAC28* as a key candidate gene for subsequent molecular and functional validation.

### 3.6. Structural and Functional Characterization of SmNAC28

**Figure 5:**
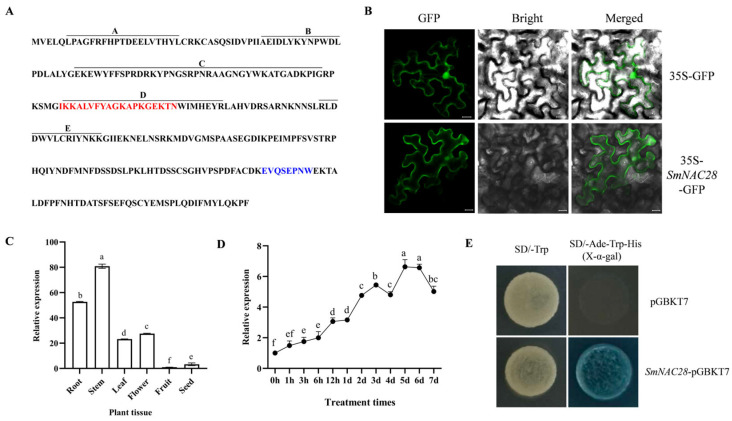
Functional characterization of *SmNAC28*. (**A**) Amino acid sequence. The nuclear localization signal contained in subdomain D of the N-terminal NAC-binding domain is indicated in red, and the sequence of the transcription activation domain specific to ATAF family members in the carboxyl-terminal region is indicated in blue. (**B**) Subcellular localization. (Scale bars = 20 μm) (**C**) Tissue-specific expression analysis. (**D**) Expression response under salt stress. (**E**) Transcriptional activation activity. Vertical bars represent the standard deviation (SD) of three biologically independent replicates (*n* = 3). Means with the same letter are not significantly different at *p* < 0.05.

Functional analysis in yeast showed that both the empty vector and *SmNAC28*-pGBKT7 transformants grew on SD/-Trp medium, but only *SmNAC28*-expressing yeast grew and turned blue on SD/-Ade/-Trp/-His medium supplemented with X-α-gal (Figure 5E). This indicates that *SmNAC28* possesses transcriptional self-activation activity. Subcellular localization experiments showed that the *SmNAC28*–GFP fusion protein localizes to both the nucleus and the plasma membrane (Figure 5B). Expression profiling indicated that *SmNAC28* is expressed in all examined tissues of eggplant, with the highest transcript levels in young stems, followed by roots (Figure 5C). Furthermore, its expression was progressively induced by 100 mM NaCl treatment, with transcript accumulation continuing to increase over time and peaking at 6 days after treatment initiation. (Figure 5D).

### 3.7. Functional Analysis of *SmNAC28* Overexpression in Enhancing Salt Tolerance

**Figure 6:**
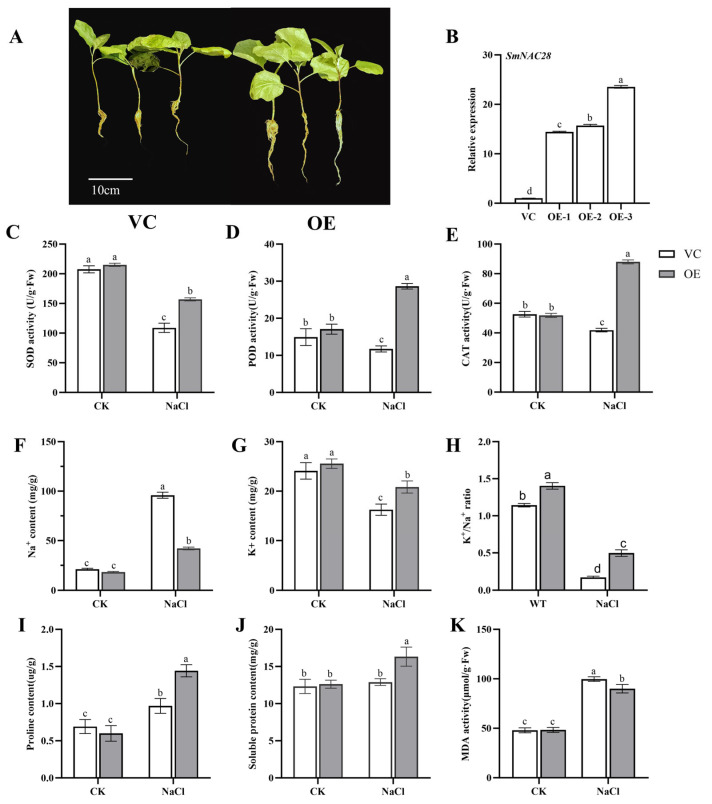
Analysis of physiological parameters between empty vector control (VC) and *SmNAC28*-overexpressing (OE) transgenic eggplant plants under control (CK) and salt stress (100 mM NaCl) conditions for 6 days. (**A**) Morphological phenotypes; (**B**) Relative expression level of *SmNAC28* in VC and OE lines; (**C**) SOD activity; (**D**) POD activity; (**E**) CAT activity; (**F**) Na^+^ content; (**G**) K^+^ content; (**H**) K^+^/Na^+^ ratio; (**I**) proline content; (**J**) soluble protein content; (**K**) MDA activity. Vertical bars represent the standard deviation (SD) of three biologically independent replicates (*n* = 3). Means with the same letter are not significantly different at *p* < 0.05.

To elucidate the role of *SmNAC28* in salt stress adaptation, we first generated transgenic composite eggplant plants overexpressing this gene. Hairy roots were induced by infecting the stem wounds of eggplant seedlings with the *Agrobacterium rhizogenes* strain K599 harboring either the *SmNAC28* overexpression construct or the empty vector (control), which were subsequently transplanted into vermiculite. PCR analysis of roots from 14 independent composite plants detected a specific amplicon, confirming the successful integration of the transgene (Figure S2). Lines OE-1 to OE-3 were selected for RT-qPCR analysis, which verified that the transcript level of *SmNAC28* in OE lines was significantly higher than that in VC (vector control) lines (Figure 6B). These three *SmNAC28*-overexpressing (OE) composite eggplant lines and empty vector control (VC) plants were subsequently subjected to functional characterization under salt stress.

Phenotypic comparison under 100 mM NaCl treatment showed that OE plants exhibited significantly enhanced salt tolerance. VC plants suffered severe growth inhibition, characterized by shorter roots, leaf wilting and chlorosis, while OE plants maintained longer roots, greener leaves, and significantly better overall growth (Figure 6A). At the physiological level, overexpression of *SmNAC28* alleviated salt-induced oxidative and osmotic damage. Under salt stress, the content of malondialdehyde (MDA), an indicator of membrane lipid peroxidation, increased sharply in VC plants but remained significantly lower in OE plants (Figure 6K). Meanwhile, after salt treatment, the activities of key antioxidant enzymes—superoxide dismutase (SOD), peroxidase (POD), and catalase (CAT)—in OE plants were significantly higher than those in VC plants (Figure 6C–E). These results indicate that *SmNAC28* overexpression enhances the ROS-scavenging capacity of transgenic eggplants, thereby reducing membrane lipid peroxidation and oxidative damage under salt stress.

In addition, under stress conditions, OE plants exhibited better ion homeostasis, accumulating less Na^+^ and more K^+^ (Figure 6F,G), and consequently maintaining a significantly higher K^+^/Na^+^ ratio (Figure 6H). Furthermore, OE plants accumulated markedly higher levels of proline and soluble protein under salt stress compared with VC plants (Figure 6I,J), which contributes to enhanced osmotic adjustment and cellular stability.

These results demonstrate that overexpression of *SmNAC28* confers enhanced salt tolerance in eggplant by reinforcing antioxidant defense, maintaining ion homeostasis, and promoting osmolyte accumulation, ultimately leading to improved growth performance under salt stress.

### 3.8. Expression Analysis of Salt-Responsive Genes in the OE and VC Hairy Roots of Composite Plants

**Figure 7:**
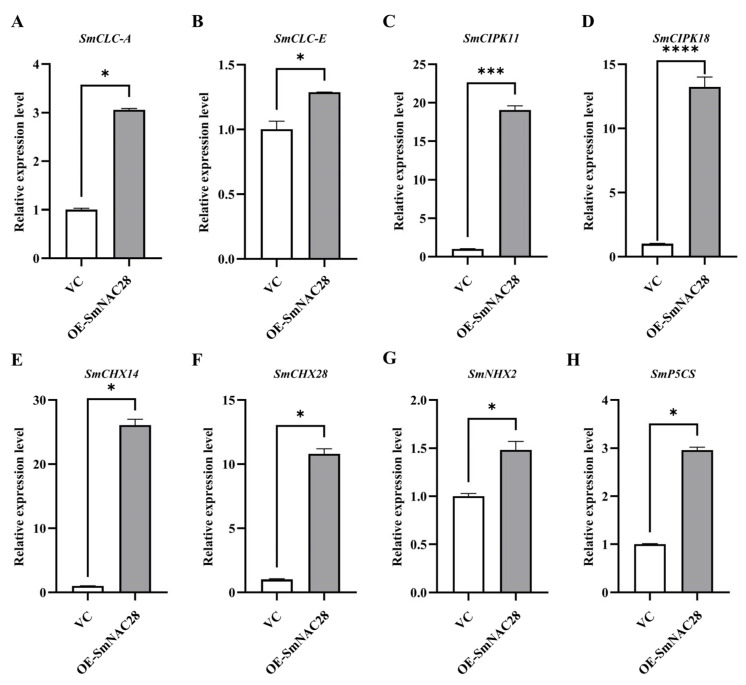
Expression level of salt-related marker genes in the hairy roots of OE and VC composite plants after salt treatment. (**A**) *SmCLC-A*, (**B**) *SmCLC-E*, (**C**) *SmCIPK11*, (**D**) *SmCIPK18*, (**E**) *SmCHX14*, (**F**) *SmCHX28*, (**G**) *SmNHX2*, and (**H**) *SmP5CS*. (*n* = 3 biologically independent repeats. two-sided Student’s *t*-test. * *p* < 0.05, *** *p* < 0.001, **** *p* < 0.0001).

Under salt stress, eggplant initiates a series of biochemical adaptive strategies to cope with salinity-induced damage. Based on transcriptome analysis, eight potential downstream target genes of NAC transcription factors were identified, including genes involved in ion transport (*SmCLCs*, *SmNHX2*, *SmCHXs*), stress signaling (*SmCIPKs*), and proline biosynthesis (*SmP5CS*). Their expression levels were analyzed by RT-qPCR in the roots of *SmNAC28*-overexpressing plants after 6 days of exposure to high salinity, and significant upregulation of these genes was observed (Figure 7). These results suggest that *SmNAC28* may directly or indirectly regulate salt stress-related genes, thereby enhancing salt tolerance in composite plants.

### 3.9. Subcellular Localization Analysis of the *SmNAC28* Protein in Eggplant

**Figure 8:**
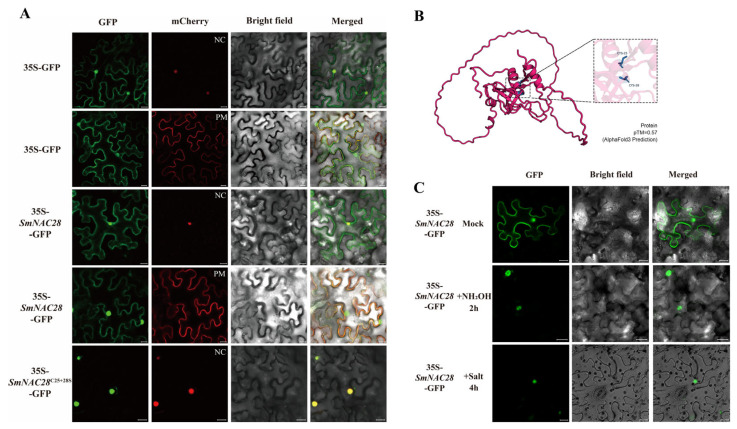
Subcellular localization and structure of *SmNAC28*. (**A**) Subcellular localization of 35S-*SmNAC28*-GFP and the double mutant 35S-*SmNAC28*^C25+28S^-GFP. The 35S-GFP construct served as the control. (Scale bars = 20 μm). Using plasma membrane (PM) and nucleus (NC) markers as reference (**B**) AlphaFold-predicted protein structure of *SmNAC28*. The overall protein structure is shown in red; the inset highlights a zoomed-in view of the region containing residues C25 and C28. (**C**) Effect of stress treatment on *SmNAC28* localization. GFP fluorescence, bright-field, and merged images of 35S-*SmNAC28*-GFP under Mock, NH_2_OH (hydroxylamine treatment), and Salt (100 mM NaCl) conditions are presented. Arrows indicate the relocalization of *SmNAC28* from the membrane to the nucleus following NH_2_OH treatment. (Scale bars = 20 μm).

As a transcription factor with transcriptional activation function, its localization on the cell membrane is often unusual (Figure 5B), which implies that it was affected by the PTMs. Prediction using CSS-Palm 4.0 software identified six potential cysteine(C) palmitoylation sites in the *SmNAC28* protein (Table S3). To validate the reliability of the AlphaFold-predicted structure, we analyzed its confidence metrics: the pLDDT score exceeded 90 for the NAC domain (residues 1–140, very high confidence), while the C-terminal transcriptional regulatory region (residues 200–283) showed low pLDDT scores (<60), typical of intrinsically disordered regions. The overall model had a pTM score of 0.57, indicating moderate global confidence (Figure S3A,B). PAE heatmap analysis showed low predicted positional errors (<5 Å) within the NAC domain but high errors (>15 Å) between the NAC domain and C-terminus, providing additional evidence for *SmNAC28*’s structural architecture, featuring a structurally ordered N-terminal domain and dynamically flexible C-terminus (Figure S3C). AlphaFold-based structural modeling further revealed that C25 and C28 are located within the conserved N-terminal CRKCASQ motif (Figure 8B, see zoomed-in view in the inset) with high confidence (pLDDT > 90). Their side chains are exposed on the protein surface, enabling sufficient accessibility for palmitoyltransferases. This CRKCASQ motif is crucial for *SmNAC28* protein membrane localization, and these structural features confirm C25 and C28 as functional S-palmitoylation sites, providing a structural basis for *SmNAC28* membrane anchorage.

The evidence of subcellular localization indicates that the wild-type *SmNAC28*-GFP fusion protein co-localized with both nuclear and plasma membrane markers, confirming its dual localization to the nucleus and plasma membrane. To investigate the function of palmitoylation, we generated site-directed mutants. When both palmitoylation sites (C25 and C28) were simultaneously mutated to Serine (S), the subcellular localization pattern of *SmNAC28*-GFP shifted exclusively to the nucleus (Figure 8A). In comparison, mutation of either single site did not alter this dual localization pattern (Figure S4), indicating that palmitoylation at both C25 and C28 is collectively required for membrane targeting of the protein.

We further examined whether environmental stress affects the localization of *SmNAC28*. After 4 h of salt stress (100 mM NaCl) treatment, the *SmNAC28*-GFP signal in tobacco leaves was predominantly observed in the nucleus (Figure 8C). Given that S-acylation is a reversible modification, we hypothesized that salt stress might induce de-acylation, leading to the dissociation of *SmNAC28* from the membrane. To test this hypothesis, we treated *SmNAC28*-GFP-expressing seedlings with 0.05 M hydroxylamine (NH_2_OH) for 2 h, a reagent that cleaves the thioester bond between the palmitate chain and the cysteine residue. As a control, seedlings were treated with ddH_2_O alone (Mock). The NH_2_OH treatment resulted in predominantly nuclear GFP signal (Figure 8C), similar to the observation under salt stress, whereas the Mock treatment showed no relocalization. In summary, these results suggest that salt stress likely promotes the dissociation of *SmNAC28* from the plasma membrane and its translocation to the nucleus through a de-acylation-dependent mechanism.

## 4. Discussion

Plants have evolved complex transcriptional regulatory networks through evolution to cope with external salt stress. As one of the largest transcription factor families in plants, NAC transcription factors play a central role in coordinating plant responses to abiotic stress. In this study, we systematically identified 101 NAC family members in eggplant and characterized a key salt-induced member, *SmNAC28*. Overexpression of *SmNAC28* significantly enhances salt tolerance in eggplant by coordinately regulating multiple pathways, including antioxidant defense, ion homeostasis, and osmotic adjustment. Furthermore, we revealed that the subcellular localization and function of *SmNAC28* are dynamically controlled by reversible S-palmitoylation, providing insights into the post-translational regulatory mechanism of this NAC transcription factor during salt stress signaling in eggplant.

In plants, the NAC transcription factor family is large and functionally diverse [6]. We identified 101 NAC members in eggplant, a number comparable to that in closely related species such as tomato (99 members [36]) and potato (110 members [37]), suggesting that NAC family size is relatively conserved within Solanaceae. Phylogenetic and collinearity analyses indicated that segmental duplication, rather than tandem duplication, is the primary driving force for the expansion of this gene family [38], a pattern also observed in tomato [25] and pear [38], implying that segmental duplication-mediated expansion may represent a common evolutionary mechanism for NAC genes in plants. Notably, *SmNAC28* and its orthologs in *Arabidopsis* (AT5G08790) and rice (Os01g0884300) exhibit a conserved upregulation pattern under salt stress, indicating that core stress-response pathways are conserved across dicots and monocots and reinforcing the functional significance of *SmNAC28* in eggplant salt tolerance.

Interestingly, the expression of *SmNAC28* peaks at 6 days after salt treatment, and this induction timing is relatively delayed compared with that of typical primary stress-responsive transcription factors, which are usually induced within a few hours. This expression pattern suggests that *SmNAC28* may act as a secondary response gene, functioning downstream of early signaling cascades to mediate sustained stress adaptation in plants. Notably, upregulation at the transcriptional level does not necessarily reflect the timing of protein activation. Our results confirm that *SmNAC28* undergoes reversible S-palmitoylation, which dynamically regulates its shuttling between the plasma membrane and the nucleus. After 4 h of salt stress treatment, deacylation triggers the rapid translocation of *SmNAC28* into the nucleus (Figure 8C), and this process may occur prior to the peak of transcript accumulation. Therefore, although transcriptional induction of *SmNAC28* may contribute to maintaining or enhancing its function under long-term stress, the early activation of the protein is mainly achieved through post-translational regulation.

The establishment of salt tolerance relies on the coordinated action of multiple genes and pathways. Our study found that overexpression of *SmNAC28* significantly improves the survival rate and growth performance of eggplant under salt stress (Figure 6A), which is closely linked to its multifaceted regulatory roles at the physiological and molecular levels. Overexpression lines showed reduced accumulation of the membrane lipid peroxidation product MDA (Figure 6K), higher activities of antioxidant enzymes (SOD, POD, CAT) (Figure 6C–E), maintained superior ion homeostasis (Figure 6F–H), and accumulated more osmoprotectant proline and elevated soluble protein content, thus contributing to improved stress tolerance. (Figure 6I,J). These physiological improvements were accompanied by the upregulation of a suite of functional genes (Figure 7), including ion transporters (*SmNHX2, SmCHXs*), stress signaling (*SmCIPKs*), and the proline biosynthesis gene *SmP5CS*. This coordinated activation of diverse downstream targets suggests that *SmNAC28* may function as an upstream transcriptional hub integrating multiple stress-responsive pathways. Such a pleiotropic regulatory mode has been observed in several NAC transcription factors, such as rice *ONAC022* [7] and soybean *GmNAC109* [13], and contrasts with NACs that primarily target single pathways, e.g., *Arabidopsis NTL8*, which mainly regulates germination without directly modulating antioxidant enzymes [12]. The ability of *SmNAC28* to simultaneously enhance antioxidant defense, ion homeostasis, and osmotic adjustment likely confers more robust and sustainable salt tolerance compared to single-pathway regulators. This pleiotropic regulatory strategy appears to be evolutionarily conserved, as recent studies in other horticultural crops further support this mechanism. For instance, the tomato NAC transcription factor *SlNAC12* enhances salt tolerance through coordinated regulation of ion homeostasis [16], antioxidant enzyme activities, and flavonoids accumulation. Similarly, overexpression of the sorghum NAC gene *SbNAC074* in tobacco significantly enhanced salt tolerance by reducing MDA and H_2_O_2_ accumulation while increasing SOD, POD, and CAT activities, demonstrating a conserved mechanism of oxidative damage mitigation [39]. Moreover, the soybean NAC transcription factor *GmNAC03* was recently shown to enhance salt tolerance by elevating antioxidant enzyme activities (SOD, POD, CAT) and modulating amino acid metabolic pathways [40]. These findings, together with our results, suggest that NAC-mediated coordination of antioxidant defense, ion homeostasis, and osmotic adjustment represents a widely conserved strategy across plant species to cope with salt stress.

Comparing *SmNAC28* with NAC transcription factors from other Solanaceous crops provides further insights into its functional context. Similar to tomato *SlNAC12* [16], *SmNAC28* enhances salt tolerance through coordinated regulation of ion homeostasis, antioxidant defense, and osmotic adjustment, suggesting that such pleiotropic NACs may serve as conserved stress-responsive hubs in Solanaceae. However, unlike tomato *SlNAC35*, which acts as a negative regulator controlled by ubiquitination [41], *SmNAC28* activity is dynamically modulated by S-palmitoylation-mediated membrane-nucleus shuttling. Potato *StNAC1* also contributes to salt tolerance via ROS and proline regulation [42], but whether it undergoes similar post-translational control remains unknown. Thus, while the core stress-responsive functions of NAC factors are broadly conserved, their regulatory mechanisms exhibit species-specific diversification.

The activity of NAC transcription factors is finely tuned not only at the transcriptional level but also by complex PTMs [19]. Among these, S-palmitoylation, a reversible lipid modification, influences their membrane association, stability, and signal transduction [43,44]. Beyond NAC family members, S-palmitoylation has been documented to regulate other membrane-associated transcription factors. For instance, the bZIP transcription factor *RSG* in tobacco undergoes S-palmitoylation at a conserved cysteine residue, which is essential for its plasma membrane localization and subsequent de-repression under gibberellin signaling [45].

In this study, we demonstrate that S-palmitoylation regulates the subcellular localization and function of *SmNAC28* in eggplant, providing a characterization of this post-translational regulatory mechanism. Subcellular localization experiments showed that wild-type *SmNAC28* localizes to both the plasma membrane and the nucleus (Figure 8A). Bioinformatics prediction combined with site-directed mutagenesis identified two cysteine residues (Cys25 and Cys28) within the NAC domain as critical palmitoylation sites (Figure 8B, Table S4). Simultaneous mutation of both sites completely abolished its membrane localization, resulting in exclusive nuclear accumulation (Figure 8A). This finding reveals a similar mechanism to that reported for MfNACsa in Medicago falcata [22] and OsNAC9 in rice [23], highlighting that S-palmitoylation-mediated regulation is a conserved feature among NAC proteins. Structural analysis revealed that these residues reside within the highly conserved CRKCASQ motif and are exposed on the protein surface (Figure 8B), providing structural accessibility for palmitoyltransferase interaction and supporting their function as S-palmitoylation targets. To further investigate the dynamic regulation of *SmNAC28*, we examined whether both salt stress and treatment with the chemical de-acylation agent hydroxylamine (NH_2_OH) triggered the dissociation of *SmNAC28* from the membrane and its translocation to the nucleus (Figure 8C). This dynamic process reveals a clear regulatory model. Under normal conditions, *SmNAC28* is anchored to the plasma membrane via S-palmitoylation, potentially in an inactive or poised state; when plants perceive salt stress signals, specific deacylases are activated, removing the palmitoyl chain and causing the protein to release from the membrane and translocate to the nucleus, thereby initiating the transcription of downstream salt-tolerance genes. This S-acylation cycle represents a rapid and reversible regulatory module that couples membrane-initiated stress perception with nuclear transcriptional reprogramming.

The reversibility of this cycle implies the involvement of specific enzymes that dynamically regulate palmitoylation status. In plants, protein S-acyl transferases (PATs), characterized by their conserved DHHC (Asp-His-His-Cys) motif, catalyze protein S-acylation and have been systematically characterized in multiple species, including *Arabidopsis*, rice, soybean [46], and more recently in woodland strawberry where 21 *FvPATs* were shown to respond to hormone and stress treatments [47]. Conversely, depalmitoylation is mediated by acyl-protein thioesterases (APTs), which remove palmitoyl moieties and facilitate protein relocalization [48]. A recent study further demonstrated that in *Medicago truncatula*, *MtPAT9* interacts with and S-acylates *MtNAC80*, while *MtAPT1* mediates its de-S-acylation under cold stress, establishing a complete S-acylation cycle that regulates stress-responsive NAC transcription factor localization and function [49]. These findings collectively indicate that the PAT-APT regulatory module represents a conserved mechanism controlling NAC protein dynamics across diverse stress conditions. Identifying the specific PAT(s) and APT(s) responsible for *SmNAC28* modification in eggplant represents an important direction for future research, which will further elucidate how this regulatory module integrates with upstream salt stress signaling pathways.

Several limitations of this study should be acknowledged. First, in addition to the need to identify the specific PAT/APT enzymes noted above, our study has other constraints. Second, although our hairy root system confirmed root specific function, generation of stable transgenic eggplant lines would enable investigation of *SmNAC28* effects on whole-plant physiology and yield under field conditions. Third, while structural analysis supports palmitoylation site accessibility, direct biochemical evidence (e.g., acyl-biotin exchange assays) would further substantiate the palmitoylation mechanism. Fourth, the regulation of downstream candidate genes (*SmNHX2*, *SmCHXs*, *SmCIPKs*, and *SmP5CS*) by *SmNAC28* is currently supported primarily by qRT-PCR correlation analysis; direct evidence of transcriptional binding, such as chromatin immunoprecipitation (ChIP-qPCR), yeast one-hybrid assays, electrophoretic mobility shift assays (EMSA), or dual-luciferase reporter assays, is required to definitively establish them as direct targets of *SmNAC28*. Addressing these questions will improve our understanding of how S-palmitoylation regulates NAC transcription factor function in eggplant and facilitate the use of *SmNAC28* in molecular breeding for salt-tolerant eggplant varieties.

## 5. Conclusions

In summary, this study systematically identified the NAC transcription factor family in eggplant and elucidated the function and regulatory mechanism of its key member, *SmNAC28*. *SmNAC28* enhances salt tolerance in eggplant, likely through its involvement in modulating antioxidant defense, ion homeostasis, and promotion of osmotic adjustment. Its function is regulated by an S-palmitoylation-mediated membrane-nucleus shuttling mechanism. Under normal conditions, palmitoylation anchors it to the cell membrane, while stress-induced deacylation triggers its nuclear translocation and the activation of downstream salt-tolerance genes. These findings not only enrich the theoretical understanding of transcriptional regulation in plant responses to salt stress but also provide a significant genetic and theoretical foundation for breeding salt-tolerant eggplant varieties through molecular breeding techniques.

## Data Availability

The original contributions presented in this study are included in the article and Supplementary Material. Further inquiries can be directed to the corresponding authors.

## Supplementary Materials

The following supporting information can be downloaded at: https://www.mdpi.com/article/10.3390/cimb48040398/s1.

## Abbreviations

The following abbreviations are used in this manuscript:

NAC	NAM, ATAF1/2, and CUC2
TF	Transcription factor
MDA	Malondialdehyde
SOD	Superoxide dismutase
POD	Peroxidase
CAT	Catalase
qRT-PCR	Quantitative real-time polymerase chain reaction
ROS	Reactive oxygen species
ABA	Abscisic acid
PTM	Post-translational modification
HMM	Hidden Markov Model
CDD	Conserved Domains Database
pI	Isoelectric point
NJ	Neighbor-Joining
MEME	Multiple Em for Motif Elicitation
MCScanX	Multiple Collinearity Scan toolkit
TBtools	Toolkit for Biologists
FPKM	Fragments Per Kilobase of transcript per Million mapped reads
CDS	Coding sequence
GFP	Green fluorescent protein
RFP	Red fluorescent protein
C	Cysteine
S	Serine
NH_2_OH	hydroxylamine
SD	Synthetic Defined
X-α-gal	5-Bromo-4-chloro-3-indolyl α-D-galactopyranoside
OE	Overexpressing
VC	Vector control
CK	Control check
ANOVA	Analysis of variance
MW	Molecular weight
GRAVY	Grand average of hydropathicity
DEGs	Differentially expressed genes
CLC	Chloride Channel Protein
NHX	Na^+^/H^+^ exchanger
CHX	cation/H+ exchanger
CIPK	Calcineurin B-like proteins interacting protein kinase
P5CS	Δ1-pyrroline-5-carboxylate synthetase
