# Supplementary material for: Identification of SmNAC28 Transcription Factor and Its Mechanism of Regulating Salt Tolerance in Eggplant via S-Palmitoylation
Source: Curr Issues Mol Biol. 2026 Apr 14;48(4):398. doi: 10.3390/cimb48040398 (PMC13114367; doi:10.3390/cimb48040398)
Supplement: Supplementary file 1 [file cimb-48-00398-s001.zip › Supplementary Materials Figures S1-S4.pdf]

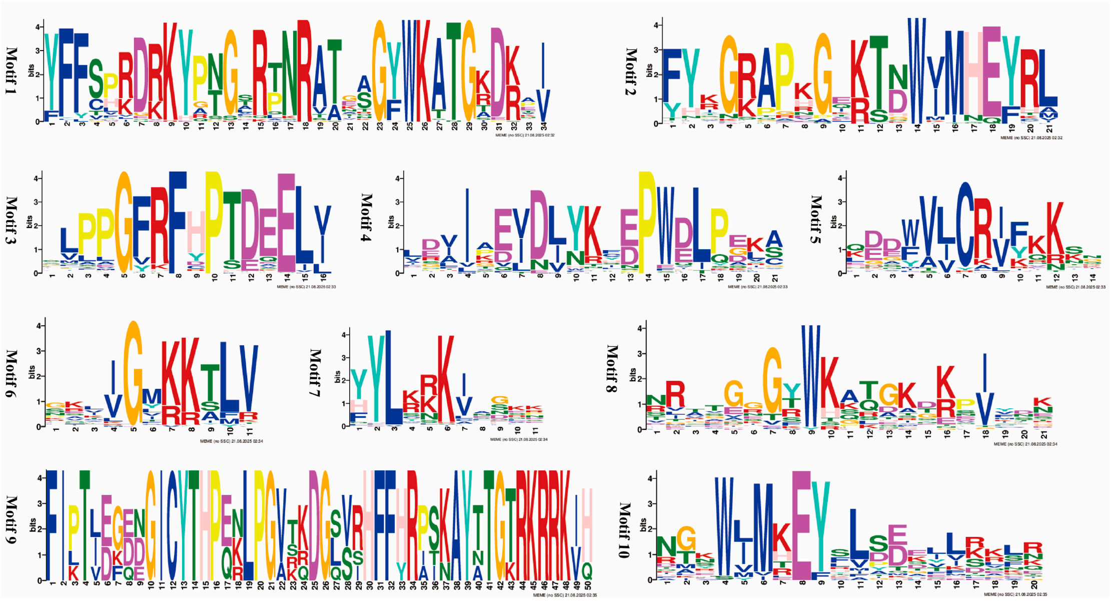

Figure S1. Conserved motif analysis of SmNAC proteins.

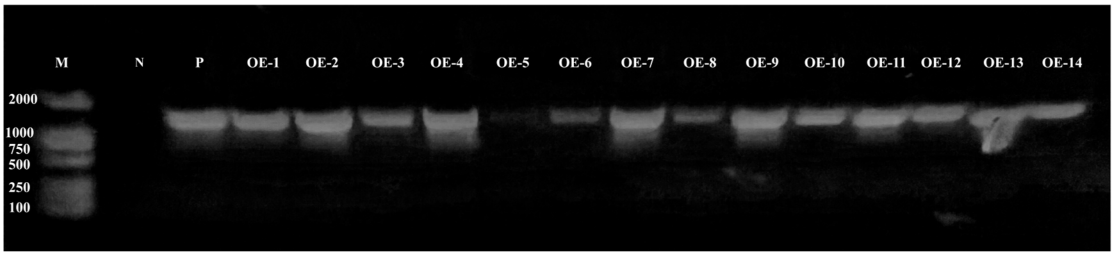

Figure S2. Detection of *SmNAC28* Gene in Transgenic Composite Eggplant Plants. (A) PCR detection of *SmNAC28* gene integration. M: DNA Marker; P: Plasmid *SmNAC28*-GFP used as a positive control; N: ddH_2O for negative control; OE-1 to 14: Independent transgenic composite eggplant lines.

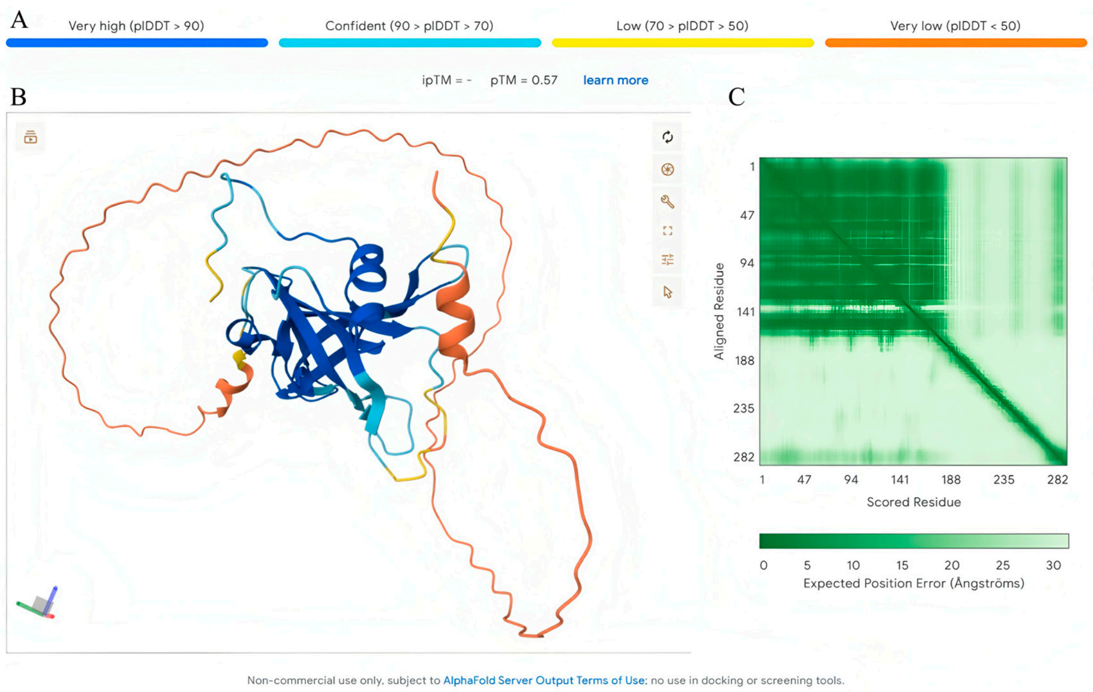

Figure S3. AlphaFold-based homology modeling and structural confidence analysis of the SmNAC28 protein. (A) Color scale for residue confidence (pLDDT). Blue (pLDDT > 90): Very high confidence; Cyan (90 > pLDDT > 70): Confident; Yellow (70 > pLDDT > 50): Low confidence; Orange (pLDDT < 50): Very low confidence. (B) Predicted three-dimensional structure of the SmNAC28 protein. Colors correspond to the pLDDT scale in panel A, visually indicating the reliability of structural prediction across different regions. (C) Predicted Aligned Error (PAE) heatmap. Both axes represent amino acid residue positions. Color intensity reflects the expected positional error (Å) between pairs of residues, with darker green indicating lower uncertainty.

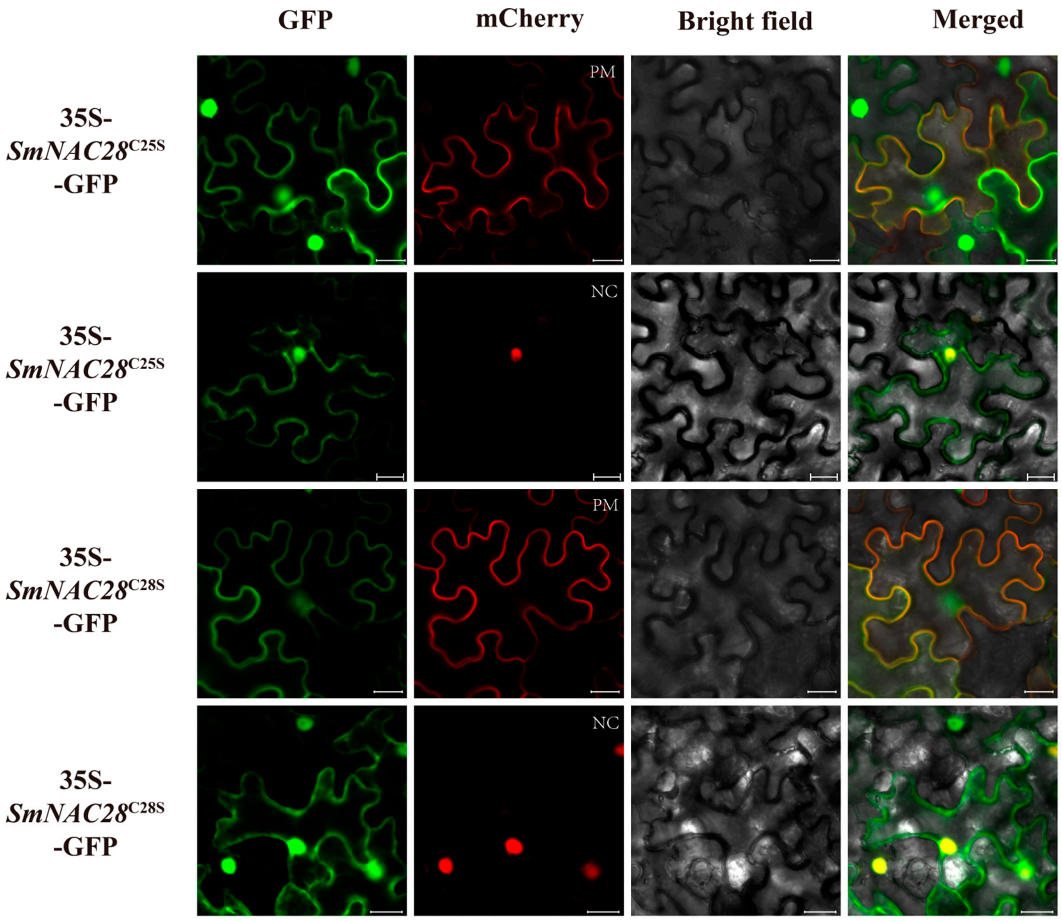

Figure S4. Subcellular localization of the *SmNAC28* single-site mutants. Scale bars = 20 μm.
