# Supplementary material for: Identification of SmNAC28 Transcription Factor and Its Mechanism of Regulating Salt Tolerance in Eggplant via S-Palmitoylation
Source: Curr Issues Mol Biol. 2026 Apr 14;48(4):398. doi: 10.3390/cimb48040398 (PMC13114367; doi:10.3390/cimb48040398)
Supplement: Supplementary file 1 [file cimb-48-00398-s001.zip › Supplementary Materials Tables S1-S4.pdf]

**Supplementary Table S1.** Primers used in this study

| Primer Name | Primer Sequence | Purposes |
|---|---|---|
| Smactin-F | GTCGGAATGGGACAGAAGGATG | Primers for qRT-PCR |
| Smactin-R | GTGCCTCAGTCAGGAGAACAGGGT | |
| SmNAC94-qPCR-F | TTCAGTTTCGACAAGACCGC | |
| SmNAC94-qPCR-R | CTGTTTTCTCCCAGTTCGGC | |
| SmCLC-A-qPCR-F | TGGCATTGGCTCCGTTACTT | |
| SmCLC-A-qPCR-R | CTCTCCAGTGCCAGGACAAG | |
| SmCLC-E-qPCR-F | CTGTCAGGTGCCTCTCACTG | |
| SmCLC-E-qPCR-R | ATTTTCCCTCTGCTCTCGGC | |
| SmCIPK11 -qPCR-F | AAGTCTCAGTCATGCGCCAA | |
| SmCIPK11 -qPCR-R | GGAGACATCCATTTCGGGCA | |
| SmCIPK18 -qPCR-F | ATTAAGCGCCTTGGCTGAGT | |
| SmCIPK18 -qPCR-R | ATAGAAGAGGCAGGCTTCGC | |
| SmCHX14-qPCR-F | GTGCAGCCTCTTTGTTGGTG | |
| SmCHX14-qPCR-R | CACCTTCCCTGCATCCACAT | |
| SmCHX28-qPCR-F | CGCATTGGTGTTTGTGACGT | |
| SmCHX28-qPCR-R | GGATCCCAACGGAACAAGGT | |
| SmNHX2-qPCR-F | TTACTGGGAGCAGCAACTGG | |
| SmNHX2-qPCR-R | TTGAGTGTGTCCCGAACGAG | |
| SmP5CS-qPCR-F | TATGCTGGCATTCCCGTTGT | |
| SmP5CS-qPCR-R | AAGAGGCCATTCCCACTTCG | |
| C-SmNAC94-F | ATGGTGGAATTGCAATTGCC | Primers for cloning |
| C-SmNAC94-R | AAATGGCTTTTGCAGATACATGAA | |
| SmNAC94-1300GFP-F | CTGCAGGGGCCCGGGGTCGACATGGTGGAATTGCAATTGCC | Primers for subcellular localization |
| SmNAC94-1300GFP-R | GCCCTTGCTCACCATGGTACCAAATGGCTTTTGCAGATACATGAA | |
| SmNAC94-TB-F | ATTTAAGTCGAAAGAGTGCATC | |

| **Primer Name** | **Primer Sequence** | **Purposes** |
|---|---|---|
| SmNAC94-TB-R | GATGCACTCTTTCGACTTAAAT | |
| SmNAC94-TB-F1 | ATTTAAGTCGAAAGTGTGCATC | |
| SmNAC94-TB-R1 | GATGCACACTTTCGACTTAAAT | |
| SmNAC94-TB-F2 | ATTTATGTCGAAAGAGTGCATC | |
| SmNAC94-TB-R2 | GATGCACTCTTTCGACATAAAT | |
| pGBKT7-SmNAC94-F | ATGGCCATGGAGGCCGAATTCATGGTGGAATTGCAATTGCC | Primers for yeast expression vector |
| pGBKT7-SmNAC94-R | CCGCTGCAGGTCGACGGATCCAAATGGCTTTTGCAGATACATGAA | |

**Supplementary Table S2.** Physicochemical Characterization of the SmNAC Transcription Factor Family

| Name | Gene ID | Amino Acids | Theoretical pI | Molecular weight | Instability index | GRAVY | Subcellular Location | Transmembrane domain |
|---|---|---|---|---|---|---|---|---|
| SmNAC1 | SMEL4.1_01g002260.1.01 | 420 | 6.66 | 47808.06 | 56.7 | -0.758 | Nucleus | 0 |
| SmNAC2 | SMEL4.1_01g008970.1.01 | 190 | 5 | 22059.48 | 55.08 | -0.741 | Nucleus | 0 |
| SmNAC3 | SMEL4.1_01g015750.1.01 | 216 | 7.25 | 25154.5 | 33.52 | -0.829 | Nucleus | 0 |
| SmNAC4 | SMEL4.1_01g035260.1.01 | 260 | 9.36 | 29940.79 | 42.64 | -0.781 | Nucleus | 0 |
| SmNAC5 | SMEL4.1_02g003960.1.01 | 554 | 5.27 | 63597.78 | 40.76 | -0.705 | Nucleus | 0 |
| SmNAC6 | SMEL4.1_02g005040.1.01 | 249 | 8.36 | 28627.82 | 41.66 | -0.542 | Nucleus | 0 |
| SmNAC7 | SMEL4.1_02g005470.1.01 | 313 | 5.69 | 35800.18 | 52.28 | -0.484 | Nucleus | 0 |
| SmNAC8 | SMEL4.1_02g013450.1.01 | 292 | 8.65 | 33358.27 | 33.46 | -0.83 | Nucleus | 0 |
| SmNAC9 | SMEL4.1_02g015790.1.01 | 294 | 8.67 | 33400.75 | 41.71 | -0.672 | Nucleus | 0 |
| SmNAC10 | SMEL4.1_02g020250.1.01 | 979 | 5.28 | 111375.13 | 36.3 | -0.449 | Chloroplast | 0 |
| SmNAC11 | SMEL4.1_02g022010.1.01 | 355 | 5.02 | 40732.46 | 40.43 | -0.754 | Nucleus | 0 |
| SmNAC12 | SMEL4.1_02g024300.1.01 | 386 | 4.89 | 43018.8 | 37.14 | -0.773 | Nucleus | 0 |
| SmNAC13 | SMEL4.1_02g028410.1.01 | 322 | 6.56 | 36747.3 | 43.3 | -0.759 | Nucleus | 0 |
| SmNAC14 | SMEL4.1_02g028660.1.01 | 401 | 6.05 | 45359.66 | 39.84 | -0.633 | Nucleus | 0 |
| SmNAC15 | SMEL4.1_03g006210.1.01 | 474 | 6.5 | 53308.29 | 45.24 | -0.836 | Nucleus | 0 |
| SmNAC16 | SMEL4.1_03g006620.1.01 | 282 | 9.03 | 32713.27 | 47.81 | -0.498 | Chloroplast | 0 |
| SmNAC17 | SMEL4.1_03g011580.1.01 | 147 | 6.59 | 16890.07 | 33.84 | -0.593 | Nucleus | 0 |
| SmNAC18 | SMEL4.1_03g015460.1.01 | 324 | 8.84 | 37698.19 | 50.47 | -0.892 | Nucleus | 0 |
| SmNAC19 | SMEL4.1_03g019550.1.01 | 735 | 5.63 | 82135.65 | 36.61 | -0.675 | Nucleus | 1 |
| SmNAC20 | SMEL4.1_03g021100.1.01 | 246 | 6.38 | 28090.7 | 54.97 | -0.601 | Nucleus | 0 |
| SmNAC21 | SMEL4.1_03g021330.1.01 | 266 | 5.46 | 30835.55 | 50.12 | -0.764 | Nucleus | 0 |
| SmNAC22 | SMEL4.1_03g021920.1.01 | 215 | 6.24 | 25208.95 | 43.16 | -0.667 | Nucleus | 0 |
| SmNAC23 | SMEL4.1_03g025930.1.01 | 289 | 6.16 | 33290.83 | 34.9 | -0.6 | Nucleus | 0 |

| Name | Gene ID | Amino Acids | Theoretical pI | Molecular weight | Instability index | GRAVY | Subcellular Location | Transmembrane domain |
|---|---|---|---|---|---|---|---|---|
| SmNAC24 | SMEL4.1_03g026910.1.01 | 330 | 7.66 | 37695.36 | 31.71 | -0.68 | Nucleus | 0 |
| SmNAC25 | SMEL4.1_03g028460.1.01 | 417 | 8.44 | 48432.91 | 30.47 | -0.662 | Nucleus | 0 |
| SmNAC26 | SMEL4.1_04g000770.1.01 | 341 | 5.76 | 39146.48 | 43.03 | -0.796 | Nucleus | 0 |
| SmNAC27 | SMEL4.1_04g002340.1.01 | 351 | 8.25 | 39331.06 | 42.75 | -0.695 | Nucleus | 0 |
| SmNAC28 | SMEL4.1_04g010980.1.01 | 283 | 6.46 | 32527.8 | 55.22 | -0.669 | Nucleus | 0 |
| SmNAC29 | SMEL4.1_04g014810.1.01 | 252 | 5.73 | 28862.43 | 42.3 | -0.653 | Nucleus | 0 |
| SmNAC30 | SMEL4.1_04g015000.1.01 | 769 | 5.24 | 86936.22 | 45.06 | -0.63 | Nucleus | 1 |
| SmNAC31 | SMEL4.1_04g019630.1.01 | 276 | 6.44 | 30435.36 | 38.76 | -0.682 | Nucleus | 0 |
| SmNAC32 | SMEL4.1_04g022630.1.01 | 501 | 6.44 | 57388.98 | 34.01 | -0.768 | Nucleus | 0 |
| SmNAC33 | SMEL4.1_05g004990.1.01 | 745 | 4.93 | 83914.57 | 39.77 | -0.469 | Nucleus | 1 |
| SmNAC34 | SMEL4.1_05g005000.1.01 | 391 | 5.41 | 43559.08 | 50.42 | -0.548 | Nucleus | 0 |
| SmNAC35 | SMEL4.1_05g006100.1.01 | 196 | 4.76 | 23167.06 | 32.67 | -0.706 | Nucleus | 0 |
| SmNAC36 | SMEL4.1_05g012210.1.01 | 317 | 5.9 | 36113.4 | 44.84 | -0.617 | Nucleus | 0 |
| SmNAC37 | SMEL4.1_05g016750.1.01 | 596 | 4.82 | 67316.93 | 50.4 | -0.539 | Nucleus | 1 |
| SmNAC38 | SMEL4.1_06g001250.1.01 | 281 | 5.88 | 32405.61 | 47.05 | -0.51 | Nucleus | 0 |
| SmNAC39 | SMEL4.1_06g010050.1.01 | 268 | 6.62 | 30884.44 | 28.66 | -0.656 | Nucleus | 0 |
| SmNAC40 | SMEL4.1_06g010630.1.01 | 285 | 6.19 | 33184.36 | 50.86 | -0.815 | Nucleus | 0 |
| SmNAC41 | SMEL4.1_06g011820.1.01 | 579 | 4.63 | 65302.5 | 38.17 | -0.642 | Nucleus | 1 |
| SmNAC42 | SMEL4.1_06g015730.1.01 | 295 | 6.51 | 33636.22 | 53.82 | -0.649 | Nucleus | 0 |
| SmNAC43 | SMEL4.1_06g016420.1.01 | 343 | 5.6 | 38768.36 | 44.62 | -0.528 | Nucleus | 0 |
| SmNAC44 | SMEL4.1_06g017780.1.01 | 278 | 5.31 | 32195.23 | 52.77 | -0.68 | Nucleus | 0 |
| SmNAC45 | SMEL4.1_06g018310.1.01 | 321 | 6.23 | 37354.69 | 49.55 | -0.853 | Nucleus | 0 |
| SmNAC46 | SMEL4.1_06g018530.1.01 | 449 | 5.85 | 51705.33 | 44.88 | -0.693 | Nucleus | 0 |
| SmNAC47 | SMEL4.1_06g018760.1.01 | 278 | 5.31 | 32190.19 | 51.83 | -0.682 | Nucleus | 0 |

| Name | Gene ID | Amino Acids | Theoretical pI | Molecular weight | Instability index | GRAVY | Subcellular Location | Transmembrane domain |
|---|---|---|---|---|---|---|---|---|
| SmNAC48 | SMEL4.1_06g018870.1.01 | 312 | 8.53 | 36103.26 | 44.48 | -0.877 | Nucleus | 0 |
| SmNAC49 | SMEL4.1_06g021160.1.01 | 222 | 5.52 | 26058.31 | 54.2 | -0.761 | Nucleus | 0 |
| SmNAC50 | SMEL4.1_06g021680.1.01 | 298 | 6.18 | 33958.96 | 40.79 | -0.656 | Nucleus | 0 |
| SmNAC51 | SMEL4.1_06g022390.1.01 | 354 | 7.6 | 39917.32 | 33.85 | -0.466 | Nucleus | 0 |
| SmNAC52 | SMEL4.1_06g024680.1.01 | 718 | 4.65 | 79557.1 | 38.64 | -0.551 | Nucleus | 0 |
| SmNAC53 | SMEL4.1_06g025360.1.01 | 325 | 9.34 | 37803.85 | 27.2 | -1.067 | Chloroplast | 0 |
| SmNAC54 | SMEL4.1_06g026140.1.01 | 407 | 9.35 | 45871.09 | 45.55 | -0.532 | Nucleus | 1 |
| SmNAC55 | SMEL4.1_06g029940.1.01 | 256 | 6.3 | 30009.8 | 47.01 | -0.698 | Chloroplast | 0 |
| SmNAC56 | SMEL4.1_07g000510.1.01 | 561 | 4.85 | 63826.03 | 40.91 | -0.693 | Cytoplasm | 0 |
| SmNAC57 | SMEL4.1_07g014300.1.01 | 71 | 5.64 | 8615.83 | 36.06 | -0.863 | Chloroplast | 0 |
| SmNAC58 | SMEL4.1_07g014310.1.01 | 247 | 9.29 | 28262.48 | 47.23 | -0.84 | Chloroplast | 0 |
| SmNAC59 | SMEL4.1_07g019890.1.01 | 272 | 9.55 | 30952.12 | 34.75 | -0.794 | Chloroplast | 0 |
| SmNAC60 | SMEL4.1_07g021850.1.01 | 129 | 9.57 | 15162.15 | 46.61 | -1.093 | Nucleus | 0 |
| SmNAC61 | SMEL4.1_07g022390.1.01 | 343 | 8.12 | 38699.47 | 43.07 | -0.558 | Nucleus | 0 |
| SmNAC62 | SMEL4.1_07g023080.1.01 | 350 | 6.97 | 39410 | 37.06 | -0.646 | Nucleus | 0 |
| SmNAC63 | SMEL4.1_07g023090.1.01 | 334 | 8.5 | 37542.14 | 43.62 | -0.752 | Nucleus | 0 |
| SmNAC64 | SMEL4.1_07g025940.1.01 | 348 | 7.17 | 39289.85 | 40.82 | -0.643 | Nucleus | 0 |
| SmNAC65 | SMEL4.1_07g026170.1.01 | 284 | 8.79 | 32829.07 | 45.67 | -0.766 | Nucleus | 0 |
| SmNAC66 | SMEL4.1_08g000700.1.01 | 345 | 5.95 | 39749.44 | 52.88 | -0.786 | Peroxisome | 0 |
| SmNAC67 | SMEL4.1_08g002750.1.01 | 313 | 9.02 | 35883.24 | 36.35 | -0.819 | Nucleus | 0 |
| SmNAC68 | SMEL4.1_08g016840.1.01 | 564 | 5.14 | 62528.37 | 39.56 | -0.637 | Nucleus | 1 |
| SmNAC69 | SMEL4.1_08g017140.1.01 | 173 | 5.33 | 19977.65 | 53.32 | -0.47 | Nucleus | 0 |
| SmNAC70 | SMEL4.1_08g017290.1.01 | 350 | 7.73 | 39347.14 | 42.86 | -0.779 | Nucleus | 0 |
| SmNAC71 | SMEL4.1_08g020560.1.01 | 339 | 5.27 | 38210.37 | 42.52 | -0.681 | Nucleus | 0 |

| Name | Gene ID | Amino Acids | Theoretical pI | Molecular weight | Instability index | GRAVY | Subcellular Location | Transmembrane domain |
|---|---|---|---|---|---|---|---|---|
| SmNAC72 | SMEL4.1_08g020750.1.01 | 387 | 6.49 | 44313.32 | 52.05 | -0.834 | Nucleus | 0 |
| SmNAC73 | SMEL4.1_08g023220.1.01 | 297 | 6.51 | 34432.52 | 53.27 | -0.826 | Nucleus | 0 |
| SmNAC74 | SMEL4.1_09g000530.1.01 | 63 | 5.13 | 7618.73 | 79.07 | -0.511 | Chloroplast | 0 |
| SmNAC75 | SMEL4.1_09g000540.1.01 | 221 | 9.58 | 25185.52 | 33.94 | 0 | Nucleus | 0 |
| SmNAC76 | SMEL4.1_09g003430.1.01 | 153 | 5.33 | 18058.25 | 64.19 | -0.775 | Nucleus | 0 |
| SmNAC77 | SMEL4.1_09g012740.1.01 | 331 | 9.31 | 37732.38 | 39.63 | -0.524 | Chloroplast | 0 |
| SmNAC78 | SMEL4.1_09g016690.1.01 | 272 | 5.77 | 31404.36 | 46.02 | -0.642 | Chloroplast | 0 |
| SmNAC79 | SMEL4.1_10g002700.1.01 | 482 | 6.17 | 55642.29 | 43.91 | -0.702 | Nucleus | 0 |
| SmNAC80 | SMEL4.1_10g003160.1.01 | 532 | 4.86 | 59857.03 | 45.03 | -0.911 | Nucleus | 0 |
| SmNAC81 | SMEL4.1_10g006990.1.01 | 316 | 6.78 | 36046.62 | 43.5 | -0.667 | Chloroplast | 0 |
| SmNAC82 | SMEL4.1_10g007440.1.01 | 172 | 8.91 | 19337.62 | 20.54 | -0.859 | Nucleus | 0 |
| SmNAC83 | SMEL4.1_10g014540.1.01 | 394 | 7.21 | 44874.4 | 47.84 | -0.651 | Nucleus | 0 |
| SmNAC84 | SMEL4.1_10g014730.1.01 | 289 | 8.29 | 33534.9 | 34.74 | -0.7 | Nucleus | 0 |
| SmNAC85 | SMEL4.1_10g022780.1.01 | 1148 | 4.38 | 126691.6 | 79.06 | -0.663 | Nucleus | 0 |
| SmNAC86 | SMEL4.1_10g026010.1.01 | 580 | 5.43 | 66346.74 | 31.95 | -0.498 | Chloroplast | 0 |
| SmNAC87 | SMEL4.1_10g026070.1.01 | 373 | 4.83 | 42197.69 | 34.84 | -0.534 | Chloroplast | 0 |
| SmNAC88 | SMEL4.1_11g001250.1.01 | 174 | 9.53 | 20593.68 | 35.82 | -0.863 | Nucleus | 0 |
| SmNAC89 | SMEL4.1_11g001270.1.01 | 232 | 8.29 | 27146.52 | 36.2 | -0.824 | Nucleus | 0 |
| SmNAC90 | SMEL4.1_11g001860.1.01 | 713 | 4.39 | 80085.49 | 34.18 | -0.571 | Nucleus | 1 |
| SmNAC91 | SMEL4.1_11g001870.1.01 | 397 | 5.29 | 44643.12 | 52.35 | -0.649 | Chloroplast | 0 |
| SmNAC92 | SMEL4.1_11g004280.1.01 | 716 | 4.66 | 79329.9 | 39.39 | -0.296 | Nucleus | 1 |
| SmNAC93 | SMEL4.1_11g016000.1.01 | 286 | 8.26 | 32909.45 | 34.93 | -0.622 | Nucleus | 0 |
| SmNAC94 | SMEL4.1_11g025690.1.01 | 302 | 7.61 | 34968.39 | 33.4 | -0.835 | Nucleus | 0 |
| SmNAC95 | SMEL4.1_12g003020.1.01 | 284 | 6.26 | 32609.28 | 34.25 | -0.451 | Cytoskeleton | 0 |

| Name | Gene ID | Amino Acids | Theoretical pI | Molecular weight | Instability index | GRAVY | Subcellular Location | Transmembrane domain |
|---|---|---|---|---|---|---|---|---|
| SmNAC96 | SMEL4.1_12g006050.1.01 | 146 | 9.12 | 17387.8 | 30.24 | -0.862 | Chloroplast | 0 |
| SmNAC97 | SMEL4.1_12g007540.1.01 | 419 | 5.06 | 48317.03 | 52 | -0.627 | Nucleus | 0 |
| SmNAC98 | SMEL4.1_12g011490.1.01 | 355 | 6.29 | 41328.42 | 48.2 | -0.941 | Nucleus | 0 |
| SmNAC99 | SMEL4.1_12g012000.1.01 | 78 | 6.32 | 9294.65 | 24.32 | -1.062 | Nucleus | 0 |
| SmNAC100 | SMEL4.1_12g015210.1.01 | 411 | 8.56 | 46565.86 | 49.26 | -0.641 | Nucleus | 1 |
| SmNAC101 | SMEL4.1_12g015440.1.01 | 259 | 8.82 | 29757.8 | 54.67 | -0.661 | Chloroplast | 0 |

**Supplementary Table S3.** Gene duplication and Ka/Ks analysis

| Gene 1 | Gene 2 | Average S-sites | Average N-sites | Ka | Ks | Ka/Ks | Duplication type | Selection Pressure | Time（MYA） |
|---|---|---|---|---|---|---|---|---|---|
| SmNAC84 | SmNAC93 | 179.9166667 | 654.0833333 | 0.142708275 | 0.634085149 | 0.225061689 | Segmental | purifying selection | 21.13617162 |
| SmNAC90 | SmNAC33 | 437.4166667 | 1572.583333 | 0.342441215 | 0.816848811 | 0.419222273 | Segmental | purifying selection | 27.22829369 |
| SmNAC100 | SmNAC54 | 258.3333333 | 920.6666667 | 0.215580548 | 0.615735414 | 0.350118806 | Segmental | purifying selection | 20.5245138 |
| SmNAC96 | SmNAC45 | 93.91666667 | 344.0833333 | 0.071077077 | 0.508194706 | 0.1398619 | Segmental | purifying selection | 16.93982355 |
| SmNAC5 | SmNAC10 | 306.3333333 | 1094.666667 | 0.194545997 | 0.734081991 | 0.265019438 | Tandem | purifying selection | 24.46939971 |
| SmNAC13 | SmNAC24 | 194.6666667 | 705.3333333 | 0.346009175 | 3.433606135 | 0.100771364 | Segmental | purifying selection | 114.4535378 |
| SmNAC13 | SmNAC51 | 194.75 | 717.25 | 0.362096763 | 1.935355201 | 0.187095766 | Segmental | purifying selection | 64.51184005 |
| SmNAC9 | SmNAC59 | 173.0833333 | 603.9166667 | 0.284134413 | 1.540448375 | 0.184449163 | Segmental | purifying selection | 51.34827917 |
| SmNAC24 | SmNAC51 | 202.0833333 | 730.9166667 | 0.164846481 | 0.604110879 | 0.272874544 | Segmental | purifying selection | 20.13702929 |
| SmNAC19 | SmNAC52 | 415.25 | 1411.75 | 0.302926558 | 0.820655246 | 0.369127669 | Segmental | purifying selection | 27.35517488 |
| SmNAC18 | SmNAC73 | 164.25 | 606.75 | 0.250584532 | 2.213675489 | 0.113198404 | Segmental | purifying selection | 73.78918297 |
| SmNAC27 | SmNAC62 | 196.75 | 700.25 | 0.464338911 | 2.541741955 | 0.182685308 | Segmental | purifying selection | 84.72473185 |
| SmNAC55 | SmNAC78 | 155.0833333 | 573.9166667 | 0.224463407 | 0.846043691 | 0.265309474 | Segmental | purifying selection | 28.20145637 |
| SmNAC38 | SmNAC78 | 163.1666667 | 619.8333333 | 0.18716175 | 0.837877142 | 0.223376126 | Segmental | purifying selection | 27.92923806 |

Note: S-Sites, number of synonymous sites; N-Sites, number of non-synonymous sites; Ka, non-synonymous substitution rate; Ks, synonymous substitution; MYA, million years ago

**Supplementary Table S4.** Prediction of Palmitoylation Sites in SmNAC28

| Position | Peptide | Score | Cutoff | Cluster |
|---|---|---|---|---|
| 25 | ELVTHYLCRKCASQS | 1.932 | 0 | S-palmitoylation:Cluster C |
| 28 | THYLCRKCASQSIDV | 1.916 | 0 | S-palmitoylation:Cluster C |
| 152 | RLDDWVLCRIYNKKG | 2.36 | 0 | S-palmitoylation:Cluster C |
| 222 | KLHTDSSCSGHVPSP | 6.459 | 0 | S-palmitoylation:Cluster A |
| 233 | VPSPDFACDKEVQSE | 1.742 | 0 | S-palmitoylation:Cluster C |
| 266 | SFSEFQSCYEMSPLQ | 5.212 | 0 | S-palmitoylation:Cluster A |
